# Characteristics and Clinical Assessment of Elbow Pain in Youth Baseball Players

**DOI:** 10.3390/sports12060161

**Published:** 2024-06-11

**Authors:** Hironobu Koseki, Shiro Kajiyama, Takayuki Shida, Iku Tomonaga, Yuta Nishiyama, Daisuke Yoshida, Satoshi Mizukami, Kazuhiro Yamaguchi, Chieko Imai

**Affiliations:** 1 Department of Health Sciences, Nagasaki University Graduate School of Biomedical Sciences, Nagasaki 852-8520, Japan; 2 Department of Orthopedic Surgery, Nagasaki University Graduate School of Biomedical Sciences, Nagasaki 852-8501, Japan; 3 Department of Physical Therapy Science, Nagasaki University Graduate School of Biomedical Sciences, Nagasaki 852-8520, Japan; 4 Department of Public Health, Nagasaki University Graduate School of Biomedical Sciences, Nagasaki 852-8523, Japan; 5 Department of Orthopedic Surgery, Yamaguchi Orthopedic Clinic, Nagasaki 850-0013, Japan

**Keywords:** youth sports, baseball, throw, elbow, pain

## Abstract

Elbow injuries related to the throwing motion in baseball during the growth period present difficulties for early detection and may cause serious future disabilities. This study aimed to comprehensively determine the characteristics and clinical assessment of elbow pain in youth baseball players. Two hundred and sixteen young baseball players underwent elbow medical examination. Basic information and physical examination, clinical assessment, and ultrasonography results were examined. Univariate analyses were statistically performed between the pain-free (98 participants) and elbow pain (118 participants) groups. The mean age was 11.6 years, and ultrasonographic abnormalities were found on the medial side of the elbow in 37.5%. In total, 118 (54.6%) participants experienced elbow pain, with 64.4% of them complaining of elbow pain on the medial side. Players with multiple positions (≥2 positions) had a significantly higher prevalence of elbow pain. Height, weight, length of baseball experience, and positive rates of horizontal flexion and moving valgus stress tests were higher in the elbow pain group than in the pain-free group. The risk factors and clinical assessment for elbow pain are useful for the prevention and early detection of throwing elbow injuries in youth baseball players.

## 1. Introduction

Baseball is one of the most popular sports, with approximately 8 million and 11.5 million baseball athletes in Japan and the United States, respectively [1,2]. However, the risk of potential injuries should not be ignored. The most significant factor for upper limb sports injury is overuse in overhead sports, and the baseball throwing motion is considered particularly problematic [3]. The shoulder and elbow most frequently experience pain, specifically among young or adolescent athletes [4,5,6]. Between 18% and 45% of youth league pitchers have experienced elbow pain or discomfort [7,8,9]. Lyman et al. [10] reported that approximately one-quarter of pitchers aged 9–12 years complained of elbow pain while pitching. Matsuura et al. [11] found that 30.5% of junior baseball players aged 7–12 years reported episodes of initial elbow pain for a year. A nationwide survey in Japan found that 12.3% of elementary school baseball players experienced elbow pain [12].

Throwing elbow injury during periods of rapid growth may cause a limited range of motion (ROM) and future disabilities [10,11,12,13]. Patients with symptoms of throwing elbow injuries usually take some time to visit a hospital; consequently, clinical examinations tend to detect a significant number of advanced injuries, making treatment more difficult [14]. Although the amount of pitching (e.g., count and frequency) is thought to be the main cause, numerous other risk factors have been hypothesized to predispose individuals to throwing elbow injuries, including age, height, years of pitching, and physical function [10,15,16,17,18,19,20,21,22]. The accumulation of small injuries from early years may cause throwing elbow injuries [21]. Therefore, the US Baseball Medical and Safety Advisory Committee has raised guidelines limiting the number of pitches [23], and the Japanese High School Baseball Federation set a limitation of 500 pitches per week in 2020. Intervention at a younger age may be ideal to prevent throwing injury.

Several studies have also investigated physical signs for throwing elbow injuries in adolescent baseball players, including altered shoulder joint ROM, increased posterior shoulder tightness, decreased grip strength, rotator cuff weakness, scapular dysfunction, lower extremity muscle tightness, and deficits in single-leg standing balance [4,17,18,24]. Matsuura et al. [11] reported that nearly 60% of players with initial elbow pain had already exhibited radiographic abnormalities (e.g., medial epicondylar fragmentation and osteochondritis dissecans of the capitellum), and they suggested that assessing the risk factors and carefully examining for throwing elbow injuries without prior elbow pain were necessary. However, few studies have examined the physical findings or signs of elbow pain in youth baseball players.

In our prefecture, regional elbow medical examinations for young baseball players are regularly held by the Nagasaki Baseball Elbow Support Network, which comprises doctors, nurses, and physiotherapists. Although previous studies have investigated throwing elbow injuries [3,13,18], to the best of our knowledge, physical and environmental factors, including trunk and lower limb flexibility, and clinical signs for the early detection of elbow pain remain controversial. The present study aimed to comprehensively determine the characteristics and the usefulness of clinical assessments for elbow pain in youth baseball players and to provide insights for early detection before it becomes severe.

## 2. Materials and Methods

### 2.1. Participants

This cross-sectional study involved youth baseball players who underwent our elbow medical examination from 2017 to 2019 and volunteered to participate. Inclusion criteria required participants to be male and between the ages of 10 and 15 years. Participants were excluded from the study if (1) they were under hospital treatment or medication, (2) they had a history of orthopedic surgery, (3) they had had an injury within the previous 6 months, or (4) they had a self-reported disability in the upper or lower extremities. A questionnaire survey was conducted regarding the players’ basic information and daily practices. The basic information included age, height, body weight, age at the start of baseball, length of baseball experience, daily practice time, practice days per week, pitching side, and main and extra positions in the most recent 1 year. The players’ position questionnaire was allowed multiple answers, including main and extra positions with overlap. Body mass index (BMI) was calculated using weight and height. We also asked players whether they complained of any pain in their elbow at the time of the survey or had experienced any episode of elbow pain in the past during throwing and requested them to identify the site of pain in the elbow (anterior, posterior, medial, or lateral). The participants were divided into two groups according to their answers: an elbow pain group, who reported pain at the time of the survey or in the past, and a pain-free group.

### 2.2. Physical Examination

A certified physical therapist (S.S., D.Y., or N.Y.) rigorously standardized and evaluated the physical function measurements. Trunk and lower limb flexibilities were evaluated using the finger-to-floor distance (FFD), straight-leg rising angle (SLR), and heel–buttock distance (HBD) [25,26,27]. The FFD, which reflects trunk flexibility, was measured with the participant standing erect on a platform 20 cm high with shoes removed and feet together. The participants were instructed to bend forward as far as possible while maintaining their knees, arms, and fingers fully extended. The vertical distance between the tip of the middle finger and the platform was measured using a supple tape measure and expressed in centimetres. The vertical distance between the platform and tip of the middle finger was positive when the participant did not reach the platform and negative when the participant could go further. The SLR, which mainly detects hamstring tightness, was assessed in the supine position, with the contralateral hip and knee fully extended along the midline and the study limb raised until the pelvis began to move. To control for pelvic tilt, the examiner held the anterior–superior iliac spine in a fixed position, while the other examiner measured the SLR angle, which is the angle between the horizontal and the axis of the raised leg. The HBD, which evaluates the flexibility of the quadriceps femoris muscle, was measured with the participants in a prone position on a mat, with their arms next to their body in a relaxed position. The examiner individually flexed the participant’s knees until the examiner felt as much resistance as possible, and the distance from the heel to the buttock was measured using a standard ruler calibrated in centimetres to the first decimal point. Three types of bilateral passive shoulder ROM, including internal rotation (IR) and external rotation (ER) in the second position (90° abduction position) and IR in the third position (90° horizontal flexion position), were measured with the participants in supine position with the elbow at 90° of flexion and the forearm in the neutral position using a standard goniometer [13,24,28]. Elbow extension (Ext) and flexion (Flex) ROM were also measured in the same supine position on an examination table. While shoulder and elbow ROM were measured, the scapula was stabilized by the examiner’s hand. The ROM of the hip was measured in the supine position, with the hip in the 90° flexion position. With the contralateral hip and knee fully extended, the hip and knee of the study limb were flexed to 90° (confirmed using a goniometer) [27,29]. While the examiner rotated the hip internally and externally to its maximal point, the other examiner measured the angle between the trunk and axis of the flexed leg.

### 2.3. Clinical Assessment

**Figure 1:**
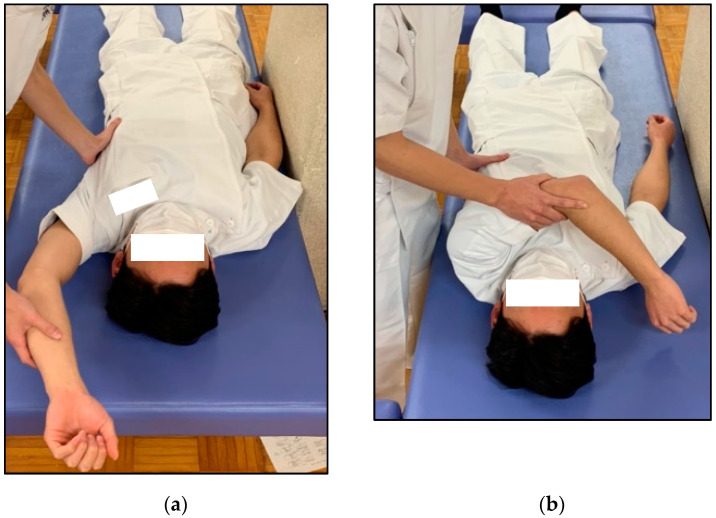
Assessments for posterior shoulder tightness. (**a**) Combined abduction test (CAT) results were considered positive if the upper arm failed to touch the head during glenohumeral abduction. (**b**) Horizontal flexion test (HFT) results were considered positive when the participant could not touch the bed beside the contralateral shoulder.

**Figure 2:**
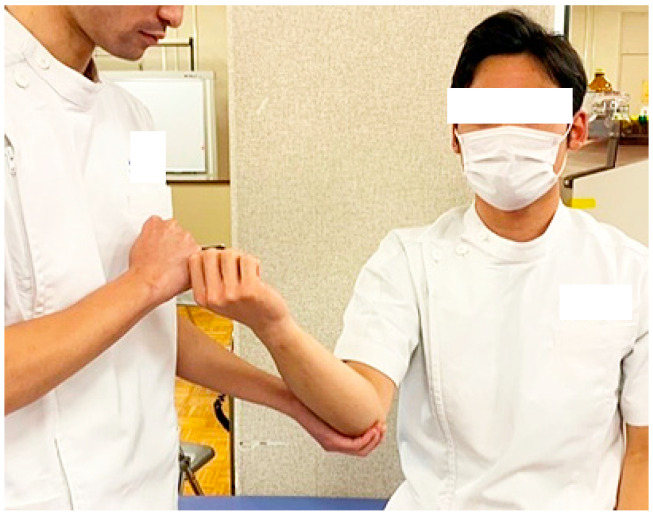
Moving Valgus Stress Test (MVST)**.** A positive sign was the presence of pain while the examiner applied and maintained a constant moderate valgus torque to the fully flexed elbow and then quickly extended the elbow.

The posterior tightness of the shoulder joint was assessed using the combined abduction test (CAT) and horizontal Flex test (HFT) in the supine position while the examiner fixed the scapula [24,30,31]. The CAT was graded as positive when the participant’s upper arm failed to touch the auricle during passive abduction of the shoulder joint (Figure 1a). Additionally, the HFT, which has the same starting position as the CAT, was determined to be positive when the participant was unable to touch the contralateral side of the bed with the fingertips during passive horizontal adduction of the shoulder joint (Figure 1b). During both examinations, the examiner completely prevented any movement of the scapula by holding it. The moving valgus stress test (MVST), a physical examination technique for the diagnosis of medial collateral ligament attenuation in the elbow, was performed [11,13,32]. The examiner applied and maintained a constant moderate valgus torque to the fully flexed elbow and then quickly extended the elbow by pulling the thumb. The presence of pain during the clinical assessment was indicated as a positive sign (Figure 2). Participants underwent a bilateral standardized measurement procedure, with the order of assessment of dominant or non-dominant.

### 2.4. Ultrasonography

Ultrasonography was performed by two authors (S.K. and K.Y.) who are experienced orthopedic surgeons with >20 years of experience in diagnosing throwing elbow injuries using an ultrasound device. Morphological conditions of the medial and lateral aspects of the dominant elbow were assessed using a 12 MHz linear-array transducer (LOGIQe, GE healthcare, Milwaukee, WI, USA). The shape of the humeral capitellum was investigated using the lateral aspect scanning in the full Ext position. The irregularity of the superficial line of the subchondral bone was classified into three stages, normal and abnormal (mild or severe), using the posterior aspect scanning in the elbow Flex position. The medial side of the elbow was evaluated for medial epicondylar fragmentation, as previously described [18,33]. The origin of the anterior transverse fibre in the medial collateral ligament was identified during elbow Flex from 70° to 90°. Irregularities or separations of the bone surfaces were classified as abnormal (mild or severe) [13].

### 2.5. Statistical Analyses

Data were assessed for normal distribution using the Shapiro–Wilk test. Continuous variables for the location of elbow pain, type of position, and number of positions held were assessed using one-way analysis of variance (ANOVA), followed by post hoc Tukey–Kramer and Bonferroni–Dunn multiple comparison tests. Univariate analyses between the pitching and non-pitching sides were performed using the chi-squared and Fisher’s exact tests for categorical variables and the unpaired *t*-test and Mann–Whitney U test for continuous variables. Furthermore, bivariate analysis was performed to compare between elbow pain group and pain-free group differences, using the unpaired *t*-test for normally distributed variables and the Mann–Whitney U test for non-normally distributed variables (i.e., height, weight, age at the start of baseball, length of experience, daily practice time, and practice days per week). The level of statistical significance was set at *p* < 0.05. We further performed a multivariate logistic regression analysis using a forward-entry method to determine the variables that were most likely associated with the occurrence of elbow pain. All data were analysed using the SPSS software (version 22.0; IBM Corp., Armonk, NY, USA). Statistical significance was defined as *p* < 0.05. A prior statistical power analysis was performed to calculate the required sample size. For a logistic regression analysis, it was determined that a total of 70 participants would be needed to detect significant differences with a statistical power of 80% [34].

### 2.6. Ethical Consideration

Institutional review board approval was obtained before the start of this retrospective cohort study, and informed consent, including the contents of this study and participants’ rights, was obtained from the parents and coaches of all participants. This study was approved by the Research Ethics Committee of Nagasaki University Graduate School of Biomedical Sciences (approval number 24011601).

## 3. Results

A total of 247 youth baseball players underwent our elbow medical examination from 2017 to 2019. None of the participants had suffered an injury within the previous 6 months or had a self-reported disability in the upper or lower extremities. We excluded 15 prospective participants with insufficient basic data, seven women, and nine under 9 years of age. Finally, 216 male youth baseball players were included. The mean age of all participants was 11.6 (range, 9–15) years. The mean height was 147.0 cm, with a mean weight of 39.7 kg and a BMI of 18.2 kg/m^2^. The mean age at the start of baseball was 8.5 years, and the average length of baseball experience was 3.0 years. The average daily practice time was 4.2 h, and the average number of practice days per week was 4.2 days. The mean FFD was 4.6 ± 3.3 cm. Of the 216 patients, 190 and 26 were right- and left-handed, respectively. The players’ positions were as follows: 98 pitchers, 50 catchers, 148 infielders, and 105 outfielders with overlaps. Ultrasonography showed that abnormalities were found on the lateral side of the elbow in six (2.8%) participants, and 80 of all 213 (37.5%) participants had mild to severe abnormal findings on the medial side, including medial epicondylar fragmentation (Table 1).

The physical findings of the pitching and non-pitching sides are shown in Table 2. No significant differences were found in the SLR, HBD, hip IR, and ER angles between the two groups. However, the pitching side showed significantly smaller values in the second and third IR angles of the shoulder joint and in the Flex and Ext angles of the elbow joint than the non-pitching side. The positive rates of the CAT were 54.4% on the pitching side and 21.3% on the non-pitching side; the positive rates of the HFT were 38.8% on the pitching side and 12.5% on the non-pitching side, whereas the positive rates of MVST were 16.7% on the pitching side and 0.9% on the non-pitching side, showing a significantly higher ratio in the pitching side than in the non-pitching side.

**Figure 3:**
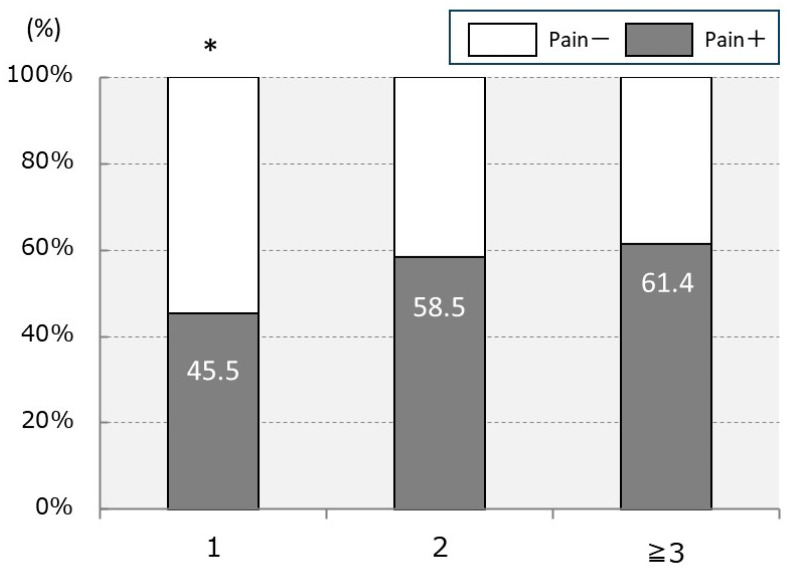
Percentage of elbow pain by the number of positions in charge. * *p* < 0.05 compared to multiple positions (≥2 positions).

Of the total, 118 (54.6%) participants had an episode of elbow pain, and all of them complained of pain on the pitching side. Among them, 38 (32.2%) participants had elbow pain at the time of survey, whereas 80 (67.8%) participants had experienced elbow pain in the past. The locations of the pain were as follows: 64.4% on the medial side of the elbow, 23.7% on the posterior side, 16.1% on the lateral side, and 6.8% on the anterior side, with the medial side being the most prevalent. In the comparison of baseball player positions (with overlap), catchers showed the highest prevalence of elbow pain at 64.0% (32/50 participants), followed by pitchers at 62.2% (61/98 participants), infielders at 57.4% (85/148 participants), and outfielders at 49.5% (52/105 participants). Although catchers and pitchers tended to experience more elbow pain than outfielders, the difference was not significant (*p* = 0.74). The incidence rates of elbow pain based on the number of positions in charge were as follows: single position, 45.5% (35/77 participants), two positions, 58.5% (55/94 participants), and three or more positions, 61.4% (27/44 participants). Players with multiple positions (≥ 2 positions) showed a significantly higher prevalence of elbow pain compared with those with a single position (*p* = 0.04) (Figure 3).

The differences in value for each variable between the pain-free (98 participants) and elbow pain (118 participants) groups (38 cases at the time of survey, 80 cases in the past) are summarized in Table 3. Univariate analysis showed that age, height, and body weight were significantly higher in the elbow pain group than in the pain-free group (*p* < 0.01). However, no significant difference was detected in BMI between the two groups. Although there were no significant differences in the items, such as the age at the start of baseball, daily practice time, and practice days per week, the elbow pain group showed significantly longer baseball experience than the pain-free group (*p* < 0.01). The values of the FFD, SLR, and ROM of the shoulder joint (second IR, second ER, and third IR) did not significantly differ between the two groups (*p* > 0.05). The HBD was higher in the elbow pain group compared to that in the pain-free group (*p* = 0.038). Regarding ROM of the elbow joint, the Ext angle in the elbow pain group (mean, 2.1 ± 4.4°) tended to be smaller than in the pain-free group (mean, 2.7 ± 5.2°); however, significant differences were not identified in either the Ext or Flex angles. Although the positive rate of the CAT was similar between the two groups, the positive rates of the HFT and MVST in the elbow pain group were significantly higher than those in the pain-free group (*p* < 0.05).

Ultrasonography revealed that abnormalities at the lateral side were observed in 1.0% (1/98 patients) of the participants in the pain-free group, whereas 4.3% (5/115 participants) of the participants in the elbow pain group had mild to severe abnormalities, including osteochondritis dissecans of the capitellum. However, the abnormal rates on medial side ultrasonography were 28.5% (28/98 participants) in the pain-free group and 45.2% (52/115 participants) in the elbow pain group, including medial epicondylar fragmentation (*p* = 0.022) (Table 4). Ultrasonography revealed no abnormalities in the non-pitching elbow.

Age, height, body weight, length of experience, HFT, and MVST, that were identified as significant factors in the univariate analysis, were used as independent variables. The MVST was significantly associated with elbow pain (*p* = 0.025; odds ratio [OR], 3.95) (Table 5).

## 4. Discussion

Repetitive throwing imparts tensile stress to the medial elbow and compressive force to the lateral elbow [35]. These forces are distributed among musculotendinous, capsuloligamentous, and bony structures. The incidence rate of elbow pain in young baseball players is approximately 20–30%, or 40–50% when history of elbow pain is included [2,10,11,12,19]. The prevalence rate was 54.6% in the present study, which is mostly consistent with these results. Regarding the locations of elbow pain, the medial side was the most common in this study, at 64.4%. Medial elbow joint space gapping and medial elbow injury occur more frequently among baseball players of all ages, from pony and little league to collegiate pitchers [3,8,9,36]. Harada et al. [18] used ultrasound imaging to investigate throwing elbow injuries in 294 baseball players (aged 9–12 years) and showed that 60 had sonographic abnormalities (medial epicondylar fragmentation in 58, and osteochondritis dissecans of the capitellum in 2).

Although there were no significant differences, pitchers and catchers tended to experience elbow pain more frequently than other players. Pitchers and catchers are thought to be at a high risk of elbow pain due to the higher amount of pitching and levels of stress they are exposed to [10,22,36]. Hattori et al. [37] reported that pitching > 60 times per session caused increased gapping of the medial elbow joint space, with a consequent increased burden on the medial elbow joint and associated tissues. Other factors, such as the performance of the slider pitch type [10], pitching with arm fatigue [16], and pitching with improper mechanics [15], also contribute, by themselves or in combination. To the best of our knowledge, no previous study has investigated the association between the number of charged positions and elbow pain. This study revealed that elbow pain was more likely to occur when multiple positions were used. This could be attributed to differences in the quality of the pitches and pitching motion required for each position, which may increase the burden on the elbow.

Regarding physical characteristics, the elbow pain group exhibited greater height and weight than the pain-free group. Higher height and annual growth rate have been considered as contributing factors for throwing elbow injuries [13,18,22]. Although the weight in the elbow pain group was significantly higher than that in the pain-free group, there was no significant difference in BMI, which suggests that weight itself may contribute to elbow pain. Greenberg et al. [38] reported that muscle and tendon growth delays relative to the rapid elongation of long tubular bones and weight gain during the growth period could cause reduced joint flexibility and hence high stress on vulnerable adolescent elbow joints, resulting in elbow pain. Nonetheless, no association was found between the flexibility of the trunk and lower limb (FFD and SLR) or ROM of the hip joint, and elbow pain. Only the HBD and the positive rate of the HFT were associated with elbow pain in this study. Decreased flexibility in the quadriceps femoris of the stance leg and limited hip IR hinder forward weight shifting and pelvic rotation from the cocking to the acceleration phase, leading to an increased load on the shoulder and elbow [17,27]. Improper coordination of the kinetic chain from the lower extremity to the trunk results in an improper upper arm position and increased forces on the elbow [39]. Considering that the participants in this study had a shorter baseball history compared with those in previous studies, it can be hypothesized that the reduction of flexibility in younger baseball players primarily occurs in the upper limbs and quadriceps femoris, and decreased flexibility of the trunk and biceps femoris, as well as limited hip IR, can be considered aggravating factors for pitching-related elbow joint disorders.

In this study, the elbow pain group tended to be older and had a longer history of playing baseball than the pain-free group. The longer the history, the heavier the repetitive rotational stress on the vulnerable growth plate around the elbow [12]. Meanwhile, no significant differences were observed in daily practice time and practice days per week. The incidence rate of elbow pain increases when the total practice time per week exceeds 14–16 h [18,22,40]. The manner and content of practice, such as warm-up exercises, basic training, base running, batting, fielding, and games, vary by team. The number and type of pitches, such as breaking balls and full-power pitches, may also affect the loading stress on the elbow joint. As a result, the amount of stress on the elbow joint may be difficult to judge based on daily practice time or practice days per week alone. Future studies should carefully evaluate the manner and content of this practice. Moreover, some teams have shorter practice sessions on weekdays and longer sessions on weekends and holidays. Therefore, it is necessary to investigate the relationship between the duration of a practice session and the occurrence of elbow pain.

Tightness of the shoulder joint, especially the decrease in the second ER angle, increases the valgus torque at the elbow joint during pitching motion, leading to throwing elbow injuries [16,18,21,28]. However, no correlation was found between the ROM in the shoulder joint, including the second ER, and elbow pain in this study, which is consistent with the findings of Sakata et al. [13]. The CAT and HFT were positive at higher rates on the throwing than on the non-throwing side, regardless of the presence of elbow pain, consistent with the findings of Shitara et al. [24]. As both the CAT and HFT are assessments of the flexibility of the glenohumeral joint, they are thought to have no direct relationship with elbow pain. However, they indicated a reduction in the extensibility of soft tissues around the shoulder joint owing to the repetition of the throwing motion. Moreover, the positive rate of the HFT was significantly higher in the elbow pain group than in the pain-free group, suggesting that tightness in the horizontal adduction of the shoulder joint may increase stress on the elbow and induce elbow pain.

No significant relationship was also observed between ROM of elbow joint and elbow pain in the present study. Repetitive, dynamic contact stress between the radial head and capitulum of the humerus during pitching motion can induce structural changes in the humeroradial joint, leading to limited ROM and pain in the elbow joint [11,41,42]. Alternatively, it has been suggested that pain-induced reflex action of the flexor muscles or increased tension of the forearm muscles due to overuse may cause a limitation of elbow extension [11,41,42]. However, these studies have mainly focused on individuals who visited hospitals with elbow pain as the main complaint; therefore, it was hypothesized that they had a relatively higher severity of impairment and lower ROM of the elbow joint compared with the participants in this study. The MVST, a pain-evoking test for medial collateral ligament injury of the elbow joint, showed a higher positive rate in the elbow pain group than in the pain-free group and was identified as the only independent related factor through logistic regression analysis. Based on these results, we conclude that the MVST is a useful assessment technique for screening early throwing injuries of the elbow.

Ultrasonography, which is a safe, inexpensive, and portable type of diagnostic imaging technique, has been used to assess throwing elbow injuries [18,33,43]. Harada et al. [33] described ultrasonography as a useful tool for detecting throwing elbow injuries early, and abnormal ultrasonographic findings were observed in 35 of 153 youth baseball players. The ultrasonographic findings of the elbow joint are thought to be related to the physical function in elementary and junior high school baseball players [18,22,33]. Therefore, it is considered an effective diagnostic test in clinical practice. In this study, ultrasonographic abnormalities were found more frequently on the medial compared to the lateral side, and the elbow pain group had a higher rate of positive abnormalities than the pain-free group. Generally, throwing elbow injuries during the growth period have a higher frequency of medial impairment [41,42,44]. In this study, 64.4% of the participants complained of elbow pain on the medial side, with a high positive rate of the MVST in the elbow pain group. When baseball players complain about the condition of the elbow joint during their growth period, it is crucial to examine medial epicondylitis injuries. Furthermore, even in the pain-free group, ultrasonographic abnormalities were observed in 1.0% on the lateral side and 28.5% on the medial side of the elbow. To anticipate the future onset of elbow pain, ultrasonography is indispensable for elbow medical examinations.

The present study has several limitations. First, we classified participants into their respective groups using self-reported recall of elbow pain. We did not require participants to have missed any time from active playing or to have any specific medical diagnosis to include them in the elbow pain group. This low threshold for allocation into the group allowed it to be heterogeneous, with some players never been specifically diagnosed with an injury. However, using this subclinical classification scheme allowed us to identify characteristics and clinical assessments that may apply to early detection, before the development of serious elbow pathological conditions. In addition, our study had a cross-sectional design and a limited number of participants, who were all male. This limited our ability to perform a proper statistical analysis of other variables. Future studies will require a more detailed analysis that includes causal relationships. An increase in the sample size, the inclusion of female participants, and a longitudinal design for the study are warranted. This may contribute to the prevention and early detection of silent throwing elbow injuries.

## 5. Conclusions

This study investigated the characteristics and clinical assessment related to elbow pain in youth baseball players. In total, 54.6% of the participants experienced elbow pain, with 64.4% of them experiencing pain on the medial side of the elbow joint on the pitching side. Higher height, heavier weight, longer experience of baseball practice, and playing multiple positions were associated with elbow pain. The MVST and ultrasonography are useful for early screening and predicting of throwing elbow injuries in youth baseball players.

## Figures and Tables

**Table 1:** Participant characteristics (N = 216).

		Mean ± SD
Age, years		11.6 ± 1.4
Height, cm		147.0 ± 11.0
Weight, kg		39.7 ± 9.4
BMI, kg/m^2^		18.2 ± 2.4
Age at the start of baseball practice, years		8.5 ± 1.4
Length of experience, years		3.0 ± 1.5
Daily practice time, hours		4.2 ± 1.8
Practice days per week, days		4.2 ± 1.5
FFD, cm		4.6 ± 3.3
		N
Pitching side (right/left)		190/26
Position ^†^	Pitcher	98
	Catcher	50
	Infielder	148
	Outfielder	105
US (N = 213)		N (%)
Lateral side	Normal	207 (97.2%)
	Mild	2 (0.9%)
	Severe	4 (1.9%)
Medial side	Normal	133 (62.4%)
	Mild	41 (19.2%)
	Severe	39 (18.3%)

Continuous variables are presented as means with standard deviations, and categorical variables are presented as numbers and percentages (%). ^†^ With overlap. BMI, body mass index; FFD, finger-to-floor distance; SD, standard deviation.

**Table 2:** Differences in variables according to pitching side.

Median (IQR)		Dominant	Non-Dominant	*p*-Value
SLR, deg		60.0 (60.0–70.0)	60.0 (60.0–70.0)	0.742
HBD, cm		5.0 (0–9.0)	6.0 (0–9.8)	0.667
Shoulder	Second IR, deg	45.0 (35.0–60.0) *	60.0 (50.0–70.0)	<0.001
	Second ER, deg	117.5 (110.0–130.0) *	110.0 (100.0–130.0)	<0.001
	Third IR, deg	30.0 (20.0–40.0) *	35.0 (30.0–45.0)	<0.001
Elbow	Flex, deg	145.0 (140.0–150.0) *	145.0 (140.0–150.0)	<0.001
	Ext, deg	0 (0–5.0) *	5.0 (0–5.0)	<0.001
Hip	IR, deg	40.0 (30.0–45.0)	40.0 (31.3–50.0)	0.089
	ER, deg	45.0 (45.0–60.0)	50.0 (45.0–60.0)	0.949
CAT +/−	N (%)	87/160 (54.4) *	34/160 (21.3)	<0.01
HFT +/−	N (%)	62/160 (38.8) *	20/160 (12.5)	<0.01
MVST +/−	N (%)	36/215 (16.7) *	2/212 (0.9)	<0.01

Continuous variables are presented as medians with IQRs, and categorical variables are presented as numbers and percentages (%). The Mann–Whitney U test was used for the continuous variables, and Pearson’s chi-squared test was used for the categorical variables. * *p* < 0.05. IQR, interquartile range; SLR, straight-leg rising; HBD, heel–buttock distance; IR, internal rotation; deg, degrees; ER, external rotation; Flex, flexion; Ext, extension; CAT, combined abduction test; HFT, horizontal flexion test, MVST, moving valgus stress test.

**Table 3:** Differences in variables according to elbow pain.

Median (IQR)		Pain-Free Group	Elbow Pain Group	*p*-Value
Age, years		11.0 (10.0–12.0) *	12.0 (11.0–13.0)	<0.01
Height, cm		143.0 (136.0–151.3) *	148.0 (142.0–157.0)	<0.01
Weight, kg		36.0 (31.3–41.0) *	40.0 (34.0–49.5)	<0.01
BMI, kg/m^2^		17.4 (16.0–19.2)	18.1 (16.7–19.8)	0.069
Age at the start of baseball practice, years		8.0 (7.0–9.0)	8.0 (8.0–9.0)	0.733
Length of experience, years		2.0 (1.0–4.0)*	3.0 (2.0–4.0)	<0.01
Daily practice time, hours		3.9 (3.0–4.7)	3.7 (2.9–4.7)	0.469
Practice days per week, days		4.0 (3.0–5.0)	4.0 (3.0–5.0)	0.827
FFD, cm		3.3 (2.3–4.6)	4.3 (1.9–7.8)	0.494
SLR (dominant), deg		60 (60–70)	60 (60–70)	0.695
HBD (dominant), cm		6.5 (2.5–10.0) *	4.0 (0.0–8.0)	0.038
Shoulder (dominant)	Second IR, deg	50 (40–60)	45 (30–51)	0.076
	Second ER, deg	120 (110–138)	115 (109–130)	0.670
	Third IR, deg	30 (20–40)	30 (20–40)	0.406
Elbow (dominant)	Flex, deg	145 (140–150)	145 (140–148)	0.235
	Ext, deg	5 (0–5)	0 (0–5)	0.231
Hip (dominant)	IR, deg	40 (35–50)	40 (30–45)	0.179
	ER, deg	50 (45–57.5)	45 (45–60)	0.380
CAT +/−	N (%)	38/75 (50.7)	49/85 (57.6)	0.376
HFT +/−	N (%)	22/75 (29.3) *	40/85 (47.1)	<0.05
MVST +/−	N (%)	7/98 (7.1) *	29/117 (24.8)	<0.01

Continuous variables are presented as medians with IQRs, and categorical variables are presented as numbers and percentages (%). The Mann–Whitney U test was used for the continuous variables, and Pearson’s chi-squared test was used for the categorical variables. * *p* < 0.05. IQR, interquartile range; BMI, body mass index; FFD, finger-to-floor distance; SLR, straight-leg rising; deg, degrees; HBD, heel-buttock distance; IR, internal rotation; ER, external rotation; Flex, flexion; Ext, extension; CAT, combined abduction test; HFT, horizontal flexion test; MVST, moving valgus stress test.

**Table 4:** Ultrasonographic abnormalities.

		Pain-Free Group	Elbow Pain Group	*p*-Value
Lateral side N (%) (N = 213)	Normal	97 (99.0%)	110 (95.7%)	0.290
	Mild	0 (0.0%)	2 (1.7%)	
	Severe	1 (1.0%)	3 (2.6%)	
Medial side N (%) (N = 213)	Normal	70 (71.4%)	63 (54.8%)	0.022 *
	Mild	17 (17.3%)	24 (20.9%)	
	Severe	11 (11.2%)	28 (24.3%)	

Categorical variables are presented as numbers and percentages (%) and Pearson’s chi-squared test was used for the categorical variables. * *p* < 0.05. Three cases considered as unclassifiable were excluded.

**Table 5:** Multivariate logistic regression models.

	Coefficient	*p*-Value	Odds Ratio	95% CI	Deviance
Age	−0.134	0.619	0.875	0.517–1.482	0.247
Height	0.045	0.222	1.046	0.973–1.123	1.494
Weight	−0.003	0.933	0.997	0.933–1.066	0.007 *
Length of experience	0.110	0.448	1.116	0.841–1.481	0.576
HFT	0.664	0.076	1.943	0.933–4.046	3.154
MVST	1.373	0.025	3.948	1.192–13.079	5.049
Constant	−5.566				

* *p* < 0.05 statistically significant. CI, confidence interval; HFT, horizontal flexion test; MVST, moving valgus stress test.

## Data Availability

The data that support the findings of this study are available from the Nagasaki Baseball Elbow Support Network, but restrictions apply to their availability, since the data were used under license for the current study and therefore are not publicly available. The data are, however, available from the authors upon reasonable request and previous permission from the Nagasaki Baseball Elbow Support Network.

## Abbreviations

ROM, range of motion; BMI: body mass index; FFD, finger-to-floor distance; SLR, straight-leg rising; HBD, heel-buttock distance; IR, internal rotation; ER, external rotation; Ext, extension; Flex, flexion; CAT, combined abduction test; HFT, horizontal flexion test; MVST, moving valgus stress test; CI, confidence interval.
